# Gut microbial histamine and serotonin promote cerebrovascular angiogenesis: Evidence from moyamoya disease

**DOI:** 10.1016/j.gendis.2026.102306

**Published:** 2026-06-13

**Authors:** Haiyan Tian, Lilan Zhu, Yuheng Zhang, Mengwei Chen, Xueying Zuo, Yi Wu, Hanzhen Wang, Zheng Lv, Qing Huang, Min Wu, Erxi Wu, Jinsheng Zeng, Xuejing Wang

**Affiliations:** a Guangdong Provincial Key Laboratory of Diagnosis and Treatment of Major Neurological Diseases, Guangzhou, Guangdong 510080, China; b National Key Clinical Department, Key Discipline of Neurology, Guangzhou, Guangdong 510080, China; c Department of Neurology, the First Affiliated Hospital of Zhengzhou University, Zhengzhou, Henan 450052, China; d Department of Neurology, the First Affiliated Hospital, Sun Yat-Sen University, Guangzhou, Guangdong 510080, China; e Hunan Clinical Research Center for Cerebrovascular Disease, Xiangya Hospital, Central South University, Changsha, Hunan 410028, China; f National Clinical Research Center for Geriatric Disorders, Xiangya Hospital, Central South University, Changsha, Hunan 410028, China; g Drug Discovery Center, Wenzhou Institute University of Chinese Academy of Sciences, Wenzhou, Zhejiang 325000, China; h Department of Critical Care Medicine, Frontiers Science Center for Disease-related Molecular Network, State Key Laboratory of Biotherapy and Cancer Center, West China Hospital, Sichuan University, Chengdu, Sichuan 610041, China; i Neuroscience Institute and Department of Neurosurgery, Baylor Scott & White Health, Temple, TX 76508, United States; j Department of Neurosurgery, Baylor College of Medicine, Temple, TX 76508, United States; k Baylor Research Institute, Baylor Scott & White Health, Temple, TX 76508, United States

**Keywords:** *Bifidobacterium pseudocatenulatum*, Cerebrovascular angiogenesis, Gut–brain axis, Histamine, Moyamoya disease, Probiotic, Serotonin, Vascular remodeling

## Abstract

Moyamoya disease (MMD) is a progressive cerebrovascular disorder characterized by arterial stenosis and compensatory neovascularization, but its upstream drivers remain poorly defined. Here, we identify *Bifidobacterium pseudocatenulatum* (*B. pseudocatenulatum*), a traditionally probiotic species, as a context-dependent pathobiont. Integrated metagenomic and metabolomic analyses revealed enrichment of *B. pseudocatenulatum* in MMD, closely associated with elevated microbial-derived histamine and serotonin. In mice, chronic oral administration of *B. pseudocatenulatum* disrupted intestinal barrier integrity and increased gut microbial production and systemic levels of histamine and serotonin. These changes were accompanied by systemic inflammation and activation of angiogenic pathways, which together promoted MMD-relevant cerebrovascular remodeling in experimental models. Notably, direct administration of histamine or serotonin to mice reproduced key MMD-like vascular phenotypes, supporting the conclusion that these microbial metabolites are sufficient to induce selected MMD-relevant vascular changes in experimental models. Our findings propose a microbial–metabolite–vascular axis and suggest that the role of specific *Bifidobacterium* strains may be more nuanced than previously recognized. These results provide a mechanistic framework for understanding the gut–brain axis in neurovascular pathology and nominate the microbiome and its metabolic products as potential targets for non-invasive diagnostics and precision therapeutic interventions in MMD.

## Introduction

Moyamoya disease (MMD) is a rare cerebrovascular disorder characterized by progressive stenosis and occlusion of the bilateral internal carotid arteries, resulting in the development of fragile, compensatory collateral vessels that resemble a “puff of smoke” on angiography.^1^ It is a leading cause of ischemic and hemorrhagic stroke in East Asian children and young adults, often resulting in severe neurological deficits, long-term disability, and death.^2^^,^^3^ Despite its substantial clinical burden, the underlying mechanisms of MMD remain poorly defined,^4^, ^5^, ^6^ and no effective pharmacological interventions currently exist to prevent disease progression.^1^^,^^7^ While surgical revascularization can improve cerebral perfusion, it is invasive, costly, and associated with significant perioperative risk.^8^ These limitations underscore the urgent need to elucidate the pathophysiology of MMD and identify novel, noninvasive therapeutic strategies.

Accumulating clinical and histological evidence suggests that immune dysregulation and vascular inflammation contribute to both the initiation and progression of MMD.^9^^,^^10^ Many patients report antecedent infections of the head, face, or throat (*e.g.*, otitis media, tonsillitis) before disease onset.^11^ Histopathological studies of occluded intracranial arteries have revealed intimal thickening, smooth muscle cell proliferation, and inflammatory cell infiltration.^12^ Elevated levels of proinflammatory mediators, including tumor necrosis factor-α (TNF-α) and cytotoxic T lymphocyte-associated antigen-4 (CTLA4), have been detected in affected vessels, supporting an immune-inflammatory etiology.^13^ However, the upstream drivers that initiate and sustain these immune and vascular abnormalities remain unknown, representing a major barrier to therapeutic development.

The gut microbiota has emerged as a powerful regulator of systemic immunity, metabolism, and vascular function.^14^ Beyond its gastrointestinal effects, gut dysbiosis has been implicated in neuroinflammation and central nervous system diseases, including stroke and neurodegenerative diseases.^15^^,^^16^ In cryptogenic and ischemic stroke, alterations in the gut microbiota have been linked to elevated systemic inflammation and poorer clinical outcomes.^17^ Stroke itself can also induce gut dysbiosis, creating a vicious cycle that amplifies brain injury.^18^ Given these bidirectional interactions, a role for the gut microbiota in MMD pathogenesis is plausible but largely unexplored. Preliminary studies have reported compositional shifts in MMD-associated microbiota,^19^^,^^20^ but these lacked mechanistic validation.

To address this critical knowledge gap, we conducted an integrated multi-omics investigation combining 16S rDNA and metagenomic sequencing of 188 fecal samples (109 MMD patients and 79 matched controls) with untargeted metabolomics profiling. This approach enabled simultaneous interrogation of microbial composition, functional potential, and metabolic output. We identified a robust disease-associated microbial signature marked by the enrichment of *Bifidobacterium pseudocatenulatum* (*B. pseudocatenulatum*), a taxon traditionally considered probiotic. This microbial expansion was closely associated with elevated fecal levels of histamine and serotonin. In mouse models, chronic oral administration of *B. pseudocatenulatum* induced gut inflammation, increased intestinal permeability, and increased circulating histamine and serotonin levels. These metabolites activated systemic inflammation (TNF-α, interleukin-1β/IL-1β) and angiogenic signaling (vascular endothelial growth factor/VEGF and endothelial nitric oxide synthase/eNOS), ultimately promoting cerebrovascular remodeling that recapitulated selected features of MMD.

Together, these findings uncover a previously unrecognized gut–brain vascular axis in MMD, wherein *B. pseudocatenulatum*-driven elevations in histamine and serotonin are associated with cerebrovascular inflammation and angiogenesis. This study supports *B. pseudocatenulatum* as a context-dependent pathobiont and highlights microbial metabolites as tractable, noninvasive targets for diagnosis and therapy in MMD.

## Materials and methods

### Ethics statement

All participants were over the age of 18 and provided written informed consent. The human microbiome study was approved by the Institutional Ethics Committees of the First Affiliated Hospital of Zhengzhou University (2021-KY-0848-002). All mouse experiments were approved by the Institutional Animal Care and Use Committee of the same institution.

### Human subjects

Patients with angiographically confirmed MMD (stage 2 or 3, per the 2012 Japanese guidelines) were enrolled between June 2022 and May 2024 from the Department of Neurosurgery or Neurology, Zhengzhou University. Digital subtraction angiography was used to confirm stenosis or occlusion of the distal internal carotid and proximal middle/anterior cerebral arteries. Of 164 screened patients, 14 with moyamoya syndrome, 36 not meeting angiographic staging, and 5 with inadequate fecal samples were excluded. The final cohort included 109 MMD patients and 79 normal controls (NC), recruited from their cohabitating spouses. Exclusion criteria included: i) active gastrointestinal disease, human immunodeficiency virus (HIV)/syphilis/hepatitis B/C, or *H. pylori* positivity; ii) recent use of antibiotics, probiotics, or proton pump inhibitors (within 3 months); iii) body mass index < 18 or > 30, abnormal lab values; iv) unhealthy lifestyle or psychiatric disorders; and v) voluntary withdrawal.

### 16S rDNA amplicon sequencing

Fecal bacterial DNA was isolated, and the V3–V4 region of the 16S rRNA gene was amplified with universal primers 341F/806R. Sequencing was performed on the Illumina Hiseq 2500 platform to generate 250 bp paired-end reads. Raw data were processed using QIIME2 (DADA2) to derive unique microbial operational taxonomic units (OTUs). Low-frequency sequences (occurrence < 5 across all samples) and duplicates were filtered out, followed by feature table construction. OTU abundance was normalized to the minimum sequencing depth per sample. Sequencing services were provided by Gene Denovo Co., Ltd., Guangzhou, China.

### Shotgun metagenomic DNA sequencing

Fecal DNA was extracted using Tiangen Biotech's Magnetic Stool DNA Kit and quantified by Qubit® 2.0 Fluorometer. Shotgun metagenomic sequencing was conducted on the Illumina NovaSeq platform, yielding an average of 20 million reads (∼6 Gb) per sample. Raw sequencing data underwent quality control, including trimming of low-quality bases (Q-score < 20) and removal of human-derived sequences via Bowtie2. Taxonomic classification was performed using DIAMOND alignment against the NCBI pan-microbial database (https://www.ncbi.nlm.nih.gov/), enabling species-level quantification across bacteria, archaea, viruses, and eukaryotes. Comparative analyses focused on species-level relative abundance. Sequencing services were provided by Novogene Biotechnology Co., Ltd., Beijing, China.

### Untargeted metabolomics analysis via UPLC-HRMS

Fecal samples from 30 MMD patients and 30 NC participants were subjected to untargeted metabolomics using ultra-high-performance liquid chromatography coupled with high-resolution mass spectrometry (UPLC-HRMS), as previously described.^21^ Briefly, fecal samples were mixed with water–methanol-acetonitrile (1:2:2), vortexed for 30 s, homogenized at 45 Hz for 4 min, sonicated at 4 °C for 5 min, and then incubated at −20 °C for 1 h. The mixtures were centrifuged at 12000 rpm at 4 °C for 10 min, and the supernatant was obtained. The fecal supernatants were submitted to the meta-Phenotyper^TM^ high coverage and precision metabolomic platform, in which three analytical methods based on UPLC-HRMS were employed to achieve comprehensive detection of metabolites and lipids. Metabolomic analyses were conducted on an Ultimate^TM^ 3000 UHPLC system coupled to a Q Exactive^TM^ quadrupole Orbitrap mass spectrometer (Thermo Scientific, San Jose, USA). The first fraction of hydrophilic extract was profiled on an ACE C_18_-PFP column (Advanced Chromatography Technologies Ltd, Aberdeen, Scotland) and detected under positive electrospray mode. The second fraction was measured on an Acquity HSS C_18_ column (Waters Corporation, Milford, USA) and detected under negative electrospray mode. Lipidomic analyses were performed on the same analytical instrument, which was operated under positive/negative polarity switching mode, and an Accucore C_30_ core–shell column (Thermo Scientific, Bellefonte, USA) was employed for chromatographic separation of lipids. Full scan mass spectra data under 70,000 FWHM resolution, as well as Top 7 or Top 10 full-scan data-dependent MS/MS spectra data, were acquired with XCalibur software (Thermo Scientific, San Jose, USA). The QC samples were evenly inserted into each data acquisition analytical batch.

### Mice

C57BL/6J mice aged 6–8 weeks were purchased from Shanghai Research Center for Southern Model Organisms (Shanghai, China). All mice were housed in an environment with constant temperature and controlled lighting (12-h/12-h light/dark cycle) and fed with rodent food and sterilized tap water. All procedures were approved by the Institutional Animal Care and Use Committee of Zhengzhou University.

### Microbe strains

*B. pseudocatenulatum* (Cat# BNCC-134343) was purchased from BeNa Culture Collection (BNCC, Shanghai, China). *B. pseudocatenulatum* was cultured in BBL medium for 72 h under anaerobic conditions. After centrifugation of the bacterial cultures, the precipitates were resuspended in sterile anaerobic phosphate-buffered saline (PBS) and measured by optical density at 600 nm (Multiskan Microplate Reader, Thermo Fisher Scientific, USA) to confirm the administered colony-forming units (CFU), and diluted with anaerobic PBS to a final concentration of 2 × 10^8^ CFU/0.2 mL.

### Preparation of different mouse models

The *B. pseudocatenulatum* group was gavaged with pure *B. pseudocatenulatum* at a dose of 2 × 10^8^ CFU per 0.2 mL suspended in sterile anaerobic PBS every 2 days. Gavage of the same dose of sterile anaerobic PBS was used as a control. Histamine (Cat# 51-45-6) and serotonin hydrochloride (serotonin, Cat# H9523) were purchased from Sigma–Aldrich (St Louis, Missouri, USA) and were dissolved in saline. The histamine was intraperitoneally injected at a dosage of 10 mg/kg, and serotonin (10 mg/kg) was intraperitoneally injected every 2 days for 12 weeks. Intraperitoneal injection of sterile PBS with the same dosing schedule was used as a control (PBS-injected mice).

### Hematoxylin–eosin staining

Hematoxylin–eosin staining was conducted in accordance with the standard procedure. Briefly, paraffin-embedded colon sections were deparaffinized, rehydrated, and stained with hematoxylin solution (Solarbio, China) for 5 min, followed by 5 dips in 1% acid ethanol (1% HCl in 75% ethanol), and then washed with distilled water. Next, the sections were stained in eosin solution (Solarbio, China) for 3 min and then washed with distilled water. After dehydration with graded alcohol and clearing in xylene, the sections were examined. The mounted slides were then examined and photographed using an Olympus IX51 microscope (Olympus, Japan).

### Immunohistochemistry staining

Deparaffinization and antigen retrieval were carried out on the 4 μm tissue sections before immunohistochemistry by boiling them in sodium citrate buffer (10 mM sodium citrate, 0.05% Tween-20, pH 6.0) at 95–100 °C for 20 min. Sections were subjected to peroxidase blocking, several PBS washes, and a 1-h incubation in a solution containing 0.5% bovine serum albumin and 3% goat serum. Then, the sections were inoculated with ZO-1 (Cat #33–9100, Invitrogen) at 4 °C overnight. The sections were cleaned in PBS and then incubated at room temperature for 1 h with a secondary antibody linked to horseradish peroxidase. The sections were then rinsed three more times for 5 min, and 3,3′-diaminobenzidine was used to stain them. Subsequently, sections were counterstained with hematoxylin to visualize nuclei. After dehydration and clearing, the sections were mounted using a permanent mounting medium. Images were captured with a BX43 upright microscope (Olympus, Japan) equipped with a DP74 camera (Olympus, Japan).

### Immunofluorescence staining

For immunofluorescence staining, the sections were rinsed in PBS and washed with PBS containing 0.5% Triton X-100 for 15 min. The sections were inoculated with the appropriate dilutions of primary antibodies, including CD31 (Cat #AF3628, R&D Systems), VEGF (Cat #Sc-7269, Santa Cruz), or α-SMA (Cat #ab21027, Abcam), in PBS at 4 °C overnight after blocking with 5% bovine serum albumin at room temperature for 30 min. Following three 5-min rinses at room temperature, the tissue slices were incubated with secondary antibodies conjugated to fluorescence at room temperature for 3 h. The tissue samples were incubated with Hoechst 33258 at a ratio of 1:1000 for 7 min after being cleaned three times with PBS at room temperature. After a second round of three 5-min room-temperature rinses, tissue slices were mounted using glycerol and glass coverslips. The preparations were kept at −20 °C until images were acquired using a BX43 Upright Microscope (Olympus, Japan) with a DP74 camera (Olympus, Japan) or a Zeiss LSM 980 (Carl Zeiss, Germany).

### Weigert's resorcin-fuchsin staining

Weigert's resorcin-fuchsin staining was conducted in accordance with the standard procedure. Briefly, paraffin-embedded artery sections were deparaffinized, rehydrated, and stained with a modified Weigert elastic fiber dye (Cat #GP1036, Servicebio, China). After dehydration with graded alcohol and clearing in xylene, the sections were examined. The mounted slides were then examined and photographed using an Olympus IX51 microscope (Olympus, Japan).

### Enzyme-linked immunosorbent assay (ELISA)

The calprotectin (CALP) and lactoferrin (LF), serotonin, histamine, IL-1β, TNF-α, VEGF, and eNOS in the mouse model were determined using mouse ELISA kits (E-EL-M1143, E-EL-M0746, E-EL-0033, E-EL-0032, E-EL-M0037, E-EL-M3063, E-EL-M1292, and E-EL-M0456 (Elabscience Biotechnology Co., Ltd., China)). First of all, Reference Standard & Sample Diluent (100 μL) was added to the blank well, and Reference Standard or test sample (100 μL) was added to the rest of the wells. Then the plate was coated with plate sealer at 37 °C for 90 min. Biotinylated Detection Ab Diluent (100 μL, prepared within 20 min before use) was applied to each well after discarding the fluid, and the plate was coated with plate sealer at 37 °C for 1 h. After liquid drainage, the plate was rinsed 3 times and soaked for 30 s each time (approximately 350 μL per well). Finally, the plate was dried by shaking and patting with absorbent paper. The prepared HRP Conjugate Diluent (100 μL, prepared within 20 min before use in a dark place) was added to each well, and the plate was coated with plate sealer at 37 °C for 30 min. Substrate reagent (TMB, 90 μL) was applied to each well, and the plate was coated with Plate Sealer at 37 °C for 15 min. Subsequently, 50 μL stop solution was added, and the absorbance of all wells was measured at 450 nm using a Multiskan Microplate Reader (Thermo Fisher Scientific, USA). The unused reagents were stored in the refrigerator at the specified temperature after the experiment was finished.

### Image quantification

For immunohistochemistry and immunofluorescence staining, quantitative analysis was performed using ImageJ software (v.1.53c bundled with 64-bit Java 1.8.0_172, US National Institutes of Health). For each mouse, three nonadjacent sections were analyzed, and three representative microscopic fields were captured from each section under identical imaging settings. Thus, nine fields per mouse were included in the quantitative analysis. Image acquisition and quantification were performed in a blinded manner, with the investigator unaware of the group allocation during image analysis. For immunohistochemistry/immunofluorescence quantification, raw images were imported into ImageJ and converted to 8-bit format when appropriate. The region of interest (ROI) corresponding to the vascular wall was manually outlined for each image. Background was subtracted uniformly, and the same threshold setting was applied to all images within the same staining batch to identify positive immunostaining. The percentage of the ROI occupied by ZO-1-, CD31-, α-SMA-, or VEGF-positive staining was then calculated as the area fraction (%). The mean value from all analyzed fields was first calculated for each section, and then the mean of the three sections was used as the final value for each mouse. For Weigert's staining measurement, three vascular cross-sections per mouse were analyzed. Images were imported into ImageJ, and two critical boundaries: i) the outer boundary of the arterial wall; and ii) the internal elastic lamina, defined as the continuous, darkly stained elastic layer. Measurements were performed at predefined points in each cross-section, and the thickness of elastic fibers (A1) and the thickness of the arterial wall (A2) were recorded. The ratio of internal elastic lamina thickness to arterial wall thickness was calculated as A1/A2 for each cross-section. For each mouse, the average value of the ratios from three independent cross-sections was taken as the final result to ensure the reliability of the data. All measurements were performed by an investigator blinded to the experimental groups. There were six mice in each group, including three males and three females.

### Statistical analysis

Qualitative data were expressed as the number of observations with the percentage, and quantitative data were expressed as mean and standard deviation, or median and interquartile range. Data from hematoxylin–eosin, immunohistochemistry, and immunofluorescence staining were analyzed by ImageJ software (US National Institutes of Health). Normality testing and tests of homogeneity of variance for all continuous variables were made before the analysis. The continuous variables between two groups were compared using Student's *t*-test, Welch's *t-*test, or two-sided Mann–Whitney *U* test according to the results of normality testing and tests of homogeneity of variance. One-way ANOVA was performed for comparison among three groups, followed by multiple comparisons test using either Tukey's or Sidak's method. Statistical significance was defined as *p* < 0.05. PERMANOVA test was performed for Bray–Curtis dissimilarity metrics using the adonis function in the R package. Linear discriminant analysis effect size (LEfSe) was used to analyze the differences in the microbial population. LDA score ≥ 3 was considered as microbes with a significant impact. For metagenome analysis, microbial relative abundance was normalized by log transformation, and the different features in each group were revealed by density ridgeline plots and box plots. For metabolomic and lipidomic data, the full scan and data-dependent MS^2^ metabolic profiles data were further processed with Compound Discoverer software (Thermo Scientific, San Jose, USA) for comprehensive component extraction. The hydrophilic metabolites were structurally annotated through searching acquired MS^2^ against a proprietary iPhenome^TM^ SMOL high-resolution MS/MS spectrum local library created using authentic standards as well as the mzCloud library (Thermo Scientific, San Jose, USA). Besides, exact *m*/*z* ± 5 ppm of MS1 spectra was searched against a local HMDB metabolite chemical database.^22^ Furthermore, retention time as well as high-resolution MS/MS spectra similarity were employed to strictly confirm the structural annotation of metabolites. The chemical identification results of metabolites and lipids were finally annotated with classification criteria proposed by Lipidomics Standardization Initiative.^23^ The metabolomic data from different measurements were first normalized using protocols described previously^24^ and then were trimmed and log transformed for further data processing. The principal component analysis, orthogonal partial least square discriminant analysis, was conducted with SIMCA software (Sartorius AG Umetrics, Goettingen, Germany). Univariate analysis, including independent sample *t*-test and false discovery rate adjusting with Benjamini and Hochberg method, ChemRICH chemical enrichment analysis,^25^ as well as data visualization, were conducted with a proprietary cloud computing platform based on R, IPOS (http://82.157.20.231:3838/ipos/). Metabolic pathway analysis of differential metabolites was conducted on the MetaboAnalyst website.^26^

## Results

### Participant demographics and integrated multi-omic study design

Initially, 121 NC and 164 individuals with MMD were screened for enrollment. Following clinical adjudication, 79 NC and 109 MMD participants—all of whom presented with angiographic stage 2 or 3 disease—met eligibility criteria and completed the study. Baseline clinical characteristics, including age, body mass index, sex, smoking and drinking history, blood pressure, serum glucose, and lipid levels, showed no significant differences between groups (Table 1). To delineate the metabolic and microbial landscapes of MMD, we employed an integrated multi-omic workflow. First, a subset of 30 NC and 30 MMD individuals was randomly selected for untargeted metabolomics to characterize fecal metabolite profiles. In parallel, fecal samples from the remaining subjects (49 NC and 79 MMD) underwent 16S rDNA sequencing to identify broad signatures of gut dysbiosis. Upon identifying significant microbial shifts at the genus level, we sought to achieve species-level resolution and evaluate functional genomic potential through shotgun metagenomic sequencing. For this high-resolution phase, we utilized a refined subset of 30 NC and 30 MMD participants. The selection of this cohort was guided by stringent quality-control requirements: shotgun metagenomic sequencing requires substantially higher DNA integrity and concentration than 16S rDNA sequencing. A total of 19 NC and 49 MMD samples were excluded because their extracted DNA did not meet strict library preparation criteria (*e.g.*, insufficient concentration or excessive fragmentation).

**Table 1:** Characteristics of study participants.

Characteristic	Normal controls (*n* = 79)	Moyamoya disease (*n* = 109)	*p value*
Age, years	44 (11.6)	46 (10.5)	0.0822
Men, *n* (%)	36 (45.6)	50 (45.9)	0.9673
Body mass index, kg/m^2^	23.93 (3.26)	23.73 (3.76)	0.684
Hypertension, *n* (%)	33 (41.8)	46 (42.2)	0.953
Diabetes, *n* (%)	7 (8.9)	20 (18.3)	0.0671
Hyperlipidemia, *n* (%)	9 (11.4)	15 (13.8)	0.6309
Smoking, *n* (%)	8 (10.1)	13 (11.9)	0.699
Drinking, *n* (%)	7 (8.9)	13 (11.9)	0.501

Note. Data are presented as mean (standard deviation) or *n* (%).

### Altered gut microbiota composition in MMD

Using 16S rDNA sequencing, we identified 634 OTUs across fecal samples from normal controls (NC, *n* = 49) and MMD patients (*n* = 79), with 95 OTUs unique to NC, 117 unique to MMD, and 422 shared between groups (Fig. 1A). Alpha diversity, assessed using the Sob and Chao 1, revealed no significant differences in microbial richness or evenness (Fig. 1B; Table S1). However, principal coordinates analysis (PCoA) based on Bray–Curtis dissimilarity demonstrated a clear separation in microbial composition between MMD and NC groups at the genus level (Fig. 1C).

**Figure 1:**
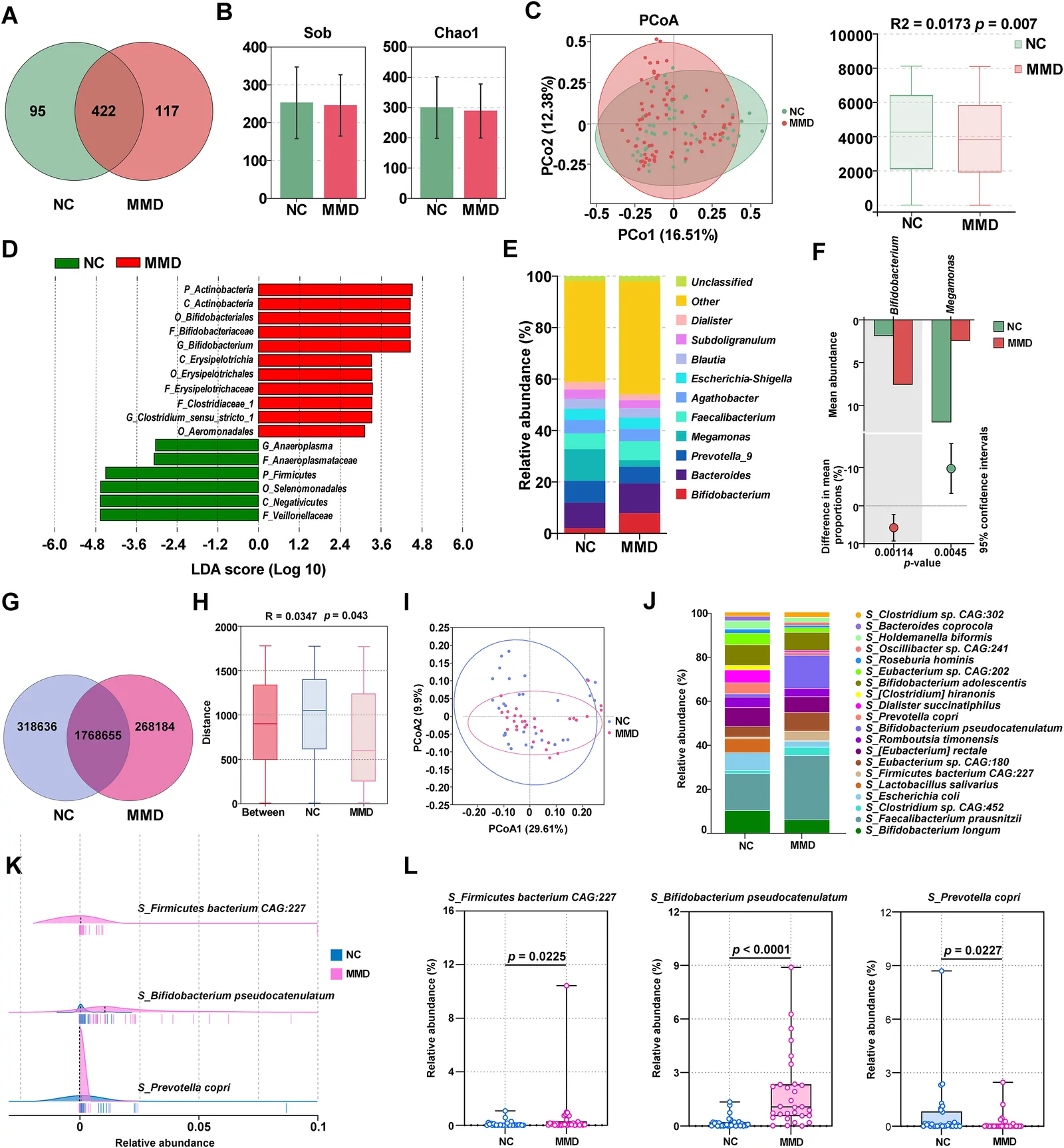
Alterations in gut microbiota composition in moyamoya disease (MMD) patients. **(A)** Venn diagram illustrating shared and unique microbial operational taxonomic units (OTUs) between normal controls (NC, *n* = 49) and MMD (*n* = 79) groups based on 16S rDNA gene sequencing. **(B)** Alpha-diversity indices (Sob and Chao1) for NC (*n* = 49) and MMD (*n* = 79) groups. Box plots display the mean with standard deviation (two-tailed Mann–Whitney *U* test). **(C)** Principal coordinates analysis (PCoA) plot based on Bray–Curtis dissimilarities of genus-level fecal microbiota from 16S rDNA gene sequencing (NC, *n* = 49; MMD, *n* = 79). Significant differentiation between groups (Adonis, *R*^2^ = 0.0173, *p* = 0.007) is indicated, with ellipses showing 95% confidence intervals. **(D)** Linear discriminant analysis effect size (LEfSe) bar chart of the LDA scores for differentially abundant bacterial taxa between NC (*n* = 49) and MMD (*n* = 79) groups (LDA score ≥ 3.0). **(E)** Relative abundance of the top 10 genera in fecal samples based on 16S rRNA gene sequencing (NC, *n* = 49; MMD, *n* = 79). **(F)** Bar plot showing differences in the relative abundance of *Bifidobacterium* and *Megamonas* between NC (*n* = 49) and MMD (*n* = 79) groups, with 95% confidence intervals (Welch's *t*-test). **(G)** Venn diagram showing the overlap of identified genes from metagenomic sequencing between NC (*n* = 30) and MMD (*n* = 30) groups. **(H)** ANOSIM analysis demonstrating significant intergroup differences in species-level microbiota composition (*r* = 0.0347, *p* = 0.043). **(I)** PCoA plot based on Bray–Curtis dissimilarities of fecal microbiota at the species level based on metagenomic sequencing (NC, *n* = 30; MMD, *n* = 30). Ellipses indicate 95% confidence intervals. **(J)** Relative abundance of the top 20 species in fecal samples based on metagenomic sequencing (NC, *n* = 30; MMD, *n* = 30). **(K)** Relative abundance distribution plots of species-level fecal microbiota from metagenomic sequencing between NC (*n* = 30) and MMD (*n* = 30) groups. **(L)** Comparison of the relative abundance distributions of *S_Firmicutes bacterium CAG:227*, *S_Bifidobacterium pseudocatenulatum*, and *S_Prevotella copri* between NC (*n* = 30) and MMD (*n* = 30) groups. The box represents the interquartile range, and the inner line denotes the median (two-tailed Mann–Whitney *U* test).

LEfSe identified taxa differentially enriched between groups. At the phylum level, *Actinobacteria* were increased, and *Firmicutes* were reduced in MMD (Fig. 1D). At the genus level, *Bifidobacterium* and *Clostridium sensu stricto 1* were markedly enriched, while *Anaeroplasma* was reduced in MMD (LDA score ≥ 3) (Fig. 1D). The 20 most abundant genera are displayed by their relative proportions (Table S2). Among the 10 most abundant genera, *Bifidobacterium* showed a marked increase while *Megamonas* was significantly reduced in MMD patients compared with NC (Fig. 1E and F).

To resolve microbial alterations at higher resolution, fecal samples from 30 NC to 30 MMD participants that passed stringent quality control were subjected to shotgun metagenomic sequencing. Gene repertoire analysis revealed 318,636 unique genes in NC and 268,184 unique genes in MMD, with 1,768,655 genes shared between the groups (Fig. 1G). Consistent with 16S rDNA results, alpha diversity based on metagenomic data showed no significant differences (Table S3). However, ANOSIM confirmed notable compositional differences at the species level (Fig. 1H), which was further supported by Bray–Curtis PCoA (Fig. 1I).

Among the top 20 most abundant species (Fig. 1J; Table S4), *B. pseudocatenulatum* and *Firmicutes bacterium CAG:227* were significantly enriched in MMD, while *Prevotella copri* was notably decreased (Fig. 1K and L). In particular, *B. pseudocatenulatum* exhibited a robust elevation in MMD (*p* < 0.001), aligning with genus-level enrichment observed in 16S rDNA sequencing. Together, these results indicate that MMD is associated with a distinct gut microbial signature, prominently characterized by the expansion of *B. pseudocatenulatum*.

### Altered fecal metabolome in patients with MMD

To investigate the functional consequences of gut dysbiosis in MMD, we performed untargeted metabolomics on fecal samples from 30 MMD patients and 30 NC. Partial least squares discriminant analysis (PLS-DA) and orthogonal PLS-DA (OPLS-DA) revealed clear separation between the metabolic profiles of MMD and NC groups (Fig. 2A and B). A 999-permutation test validated the robustness of the OPLS-DA model, indicating no significant overfitting (Fig. 2C).

**Figure 2:**
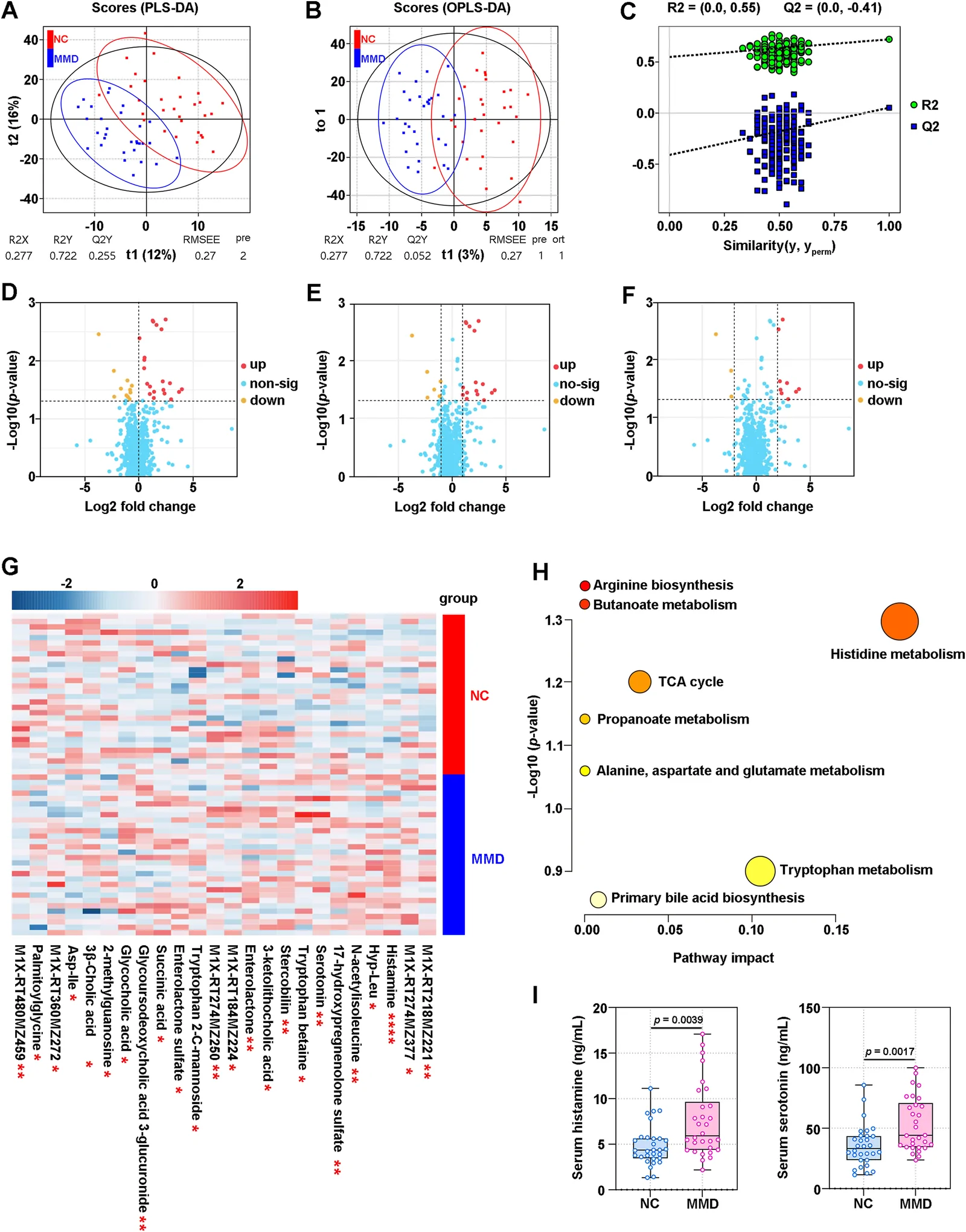
Aberrant fecal metabolic profiles in moyamoya disease (MMD) patients. **(A)** Scores plot of partial least squares discriminant analysis (PLS-DA) demonstrating separation between NC (*n* = 30) and MMD (*n* = 30) fecal samples. Each point represents an individual sample, with colored ellipses indicating 95% confidence intervals. **(B)** Scores plot of orthogonal partial least squares discriminant analysis (OPLS-DA) showing enhanced separation between NC (*n* = 30) and MMD (*n* = 30) groups. **(C)** Validation plot assessing OPLS-DA model robustness. The green circles represent R^2^ values, and the blue squares denote Q^2^ values; the dashed lines indicate similarity per permutation. **(D)** Volcano plot illustrating differential metabolites between NC (*n* = 30) and MMD (*n* = 30) groups. The red indicates up-regulated, the yellow indicates down-regulated, and the blue indicates non-significant metabolites. **(E)** Volcano plot highlighting differential metabolites. Fold-change values ≥ 1.0 were used to indicate directionality (up-regulated *vs*. down-regulated), and statistical significance was defined as *p* < 0.05. The red indicates up-regulated, the yellow indicates down-regulated, and the blue indicates non-significant metabolites. **(F)** Volcano plot highlighting differential metabolites. Thresholds of fold change ≥ 2.0 and *p* < 0.05 were applied. The red indicates up-regulated, the yellow indicates down-regulated, and the blue indicates non-significant metabolites. **(G)** Heatmap displaying relative expression levels of significantly altered metabolites (VIP > 1.00, *p* < 0.04) across NC (*n* = 30) and MMD (*n* = 30) groups. **(H)** Metabolic pathway analysis of differential metabolites (*p* < 0.05 of *t*-test) between NC (*n* = 30) and MMD (*n* = 30) groups, using the over-representation method in the MetaboAnalyst website. The horizontal axis in the figure represents the pathway impact, where a larger bubble radius indicates a greater pathway impact. The vertical axis shows the significance level of the over-representation analysis results, with colors indicating pathway significance levels; deeper colors represent higher significance levels (lower *p*-values). **(I)** Serum histamine and serotonin between NC (*n* = 30) and MMD (*n* = 30). The box represents the interquartile range, and the inner line denotes the median (two-tailed Mann–Whitney *U* test). *∗p* < 0.05, *∗∗p* < 0.01, and *∗∗∗p* < 0.001; non-sig, not significant. TCA, tricarboxylic acid.

Among the 772 metabolites analyzed, 33 were substantially altered in MMD compared with NC (Fig. 2D). Using fold change value ≥ 1.0 to determine directionality, we identified 22 differentially abundant metabolites, including 16 up-regulated and 6 down-regulated metabolites (Fig. 2E). Applying a more stringent cutoff (fold-change ≥ 2.0), 12 metabolites remained significantly dysregulated, comprising 9 up-regulated and 3 down-regulated metabolites (Fig. 2F).

A heatmap visualization of metabolites with variable importance in projection (VIP) scores > 1.0 and *p*-values < 0.04 further highlighted key differentials (Fig. 2G). Of these, glycoursodeoxycholic acid 3-glucuronide, serotonin, tryptophan betaine, M1X-RT274MZ377, M1X-RT184MZ224, M1X-RT274MZ250, histamine, succinic acid, 3-ketolithocholic acid, glycocholic acid, Hyp-Leu, enterolactone sulfate, enterolactone, stercobilin, M1X-RT218MZ221, 17-hydroxypregnenolone sulfate, and N-acetylisoleucine were up-regulated in the MMD group. In contrast, palmitoylglycine, Asp–Ile, 2-methylguanosine, 3β-Cholic acid, M1X-RT360MZ272, tryptophan 2-C-mannoside, and M1X-RT480MZ459 were down-regulated in the MMD group (Fig. 2G). Furthermore, we measured serum histamine and serotonin levels using ELISA, and found that circulating levels of histamine and serotonin were significantly increased in the MMD group (Fig. 2I). Metabolic pathway analysis based on the differential metabolites (*p* < 0.05, *t*-test) between NC and MMD groups using an over-representation method showed that a total of eight pathways were enriched, including arginine biosynthesis (*p* = 0.044), butanoate metabolism (*p* = 0.048), histidine metabolism (*p* = 0.051), tricarboxylic acid (TCA) cycle (*p* = 0.063), propanoate metabolism (*p* = 0.072), alanine, aspartate and glutamate metabolism (*p* = 0.087), tryptophan metabolism (*p* = 0.13), and primary bile acid biosynthesis (*p* = 0.14). Among these, arginine biosynthesis and butanoate metabolism were significantly enriched (Fig. 2H). Although histidine metabolism and tryptophan metabolism did not meet the threshold for statistical significance at the pathway level, they were biologically prioritized because they exhibited relatively high pathway impact scores and were characterized by marked alterations in key bioactive metabolites—histamine and serotonin—which are well-established regulators of vascular tone, endothelial activation, and neurovascular signaling. Given the established roles of these metabolites in vascular regulation and neurovascular signaling, these pathways were biologically prioritized for mechanistic exploration.

Although host cells can also produce these metabolites, their enrichment in fecal metabolomes, together with our chronic oral administration experiments, supports a substantial microbial contribution to these metabolites in MMD, with additional contribution from host pathways. Collectively, these findings suggest that gut dysbiosis contributes to MMD pathogenesis by modulating host neurovascular signaling through microbial metabolites.

### *B. pseudocatenulatum*-derived metabolites promote an MMD-relevant angiogenic phenotype

To evaluate the causal relationship between *B. pseudocatenulatum* and MMD-relevant pathology, we gavaged C57BL/6J mice with *B. pseudocatenulatum* or sterile PBS every other day for 24 weeks (Fig. 3A). At 24 weeks post-first gavage (wpfg), hematoxylin–eosin staining revealed pronounced inflammatory cell infiltration in the colonic mucosa of *B. pseudocatenulatum*-treated mice, which was absent in PBS controls (Fig. 3B). Immunohistochemical analysis showed a marked reduction in ZO-1 expression in *B. pseudocatenulatum*-treated mice, indicating disruption of intestinal epithelial barrier integrity (Fig. 3C). Correspondingly, fecal levels of CALP and LF, markers of gut inflammation and permeability, were significantly elevated in *B. pseudocatenulatum*-treated mice at 12 and 24 wpfg, but not at 4 wpfg (Fig. 3D).

**Figure 3:**
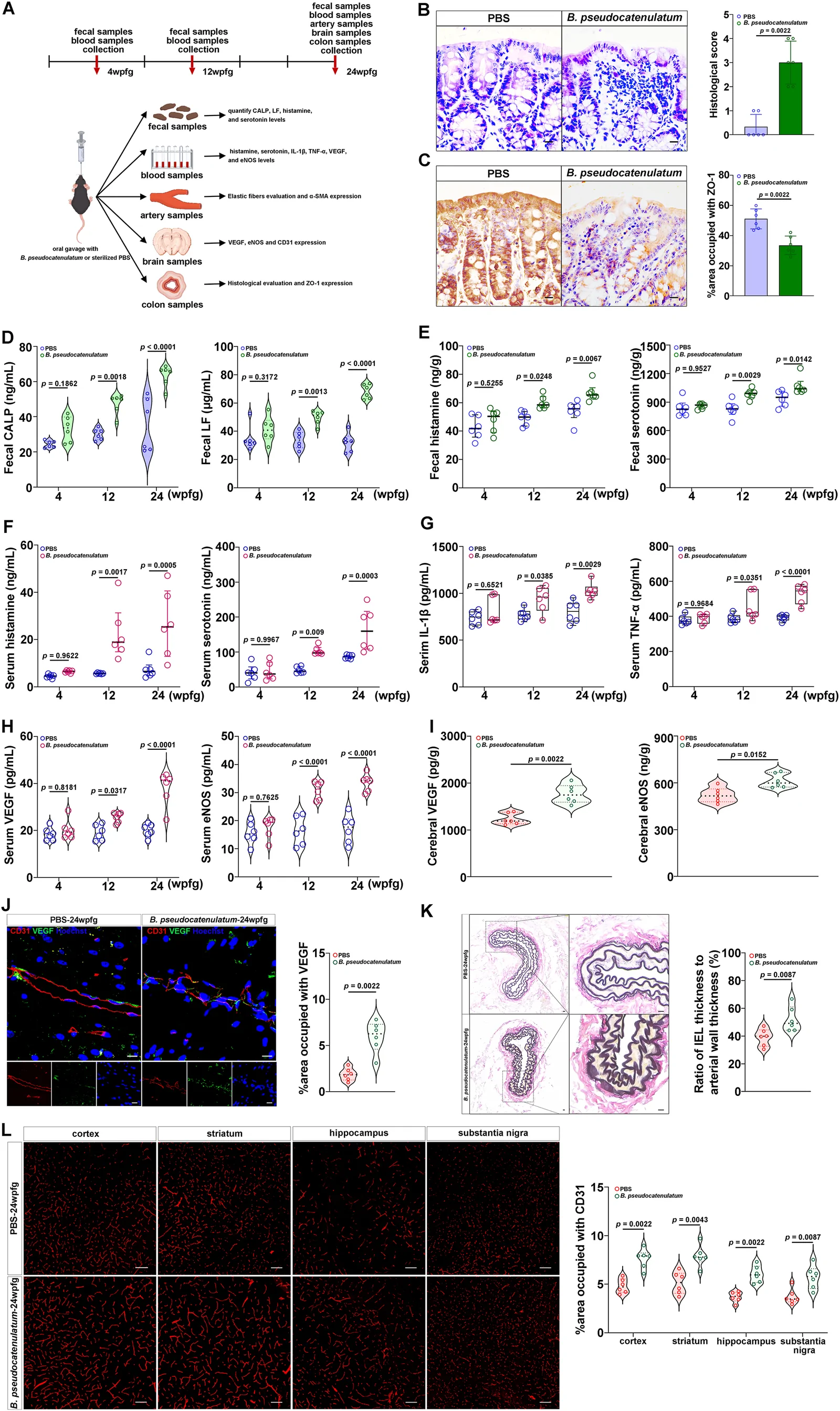
Chronic *Bifidobacterium pseudocatenulatum* administration induces intestinal dysfunction and moyamoya disease-relevant vasculopathy. **(A)** Schematic of the experimental design for *B. pseudocatenulatum* administration in C57BL/6J mice. **(B)** Representative hematoxylin–eosin-stained images of colon tissue (left) and quantification of histological scores (right) from PBS control mice and *B. pseudocatenulatum* mice at 24 wpfg. Scale bar, 20 μm. Data were shown as mean ± standard deviation with *p* values (Wilcoxon rank-sum test). *n* = 6 mice/group. **(C)** Representative immunohistochemical images (left) and quantification of % area occupied for ZO-1 (right) in colon tissue from PBS-treated and *B. pseudocatenulatum*-treated mice at 24 wpfg. Scale bar, 20 μm. Data were shown as mean ± standard deviation with *p* values (Mann–Whitney *U* test). *n* = 6 mice/group. **(D**–**G)** ELISA analysis of fecal CALP and LF (D), fecal histamine and serotonin (E), serum histamine and serotonin (F), and serum IL-1β and TNF-α (G) between PBS-treated and *B. pseudocatenulatum*-treated mice at 4, 12, and 24 wpfg. All box-and-whisker plots depict the median, quartiles, and range (two-way ANOVA with Tukey's multiple comparisons test). *n* = 6 mice/group. **(H)** ELISA analysis of serum VEGF and eNOS levels between PBS-treated and *B. pseudocatenulatum*-treated mice at 4, 12, and 24 wpfg. All box-and-whisker plots depict the median, quartiles, and range (two-way ANOVA with Tukey's multiple comparisons test). *n* = 6 mice/group. **(I)** ELISA analysis of cerebral VEGF and eNOS levels between PBS-treated and *B. pseudocatenulatum*-treated mice at 24 wpfg. All box-and-whisker plots depict the median, quartiles, and range (Mann–Whitney *U* test). *n* = 6 mice/group. **(J)** Representative immunofluorescence images of VEGF (green) and CD31 (red) in brain sections (left), and quantification of VEGF area % (right) between PBS-treated and *B. pseudocatenulatum*-treated mice at 24 wpfg. Scale bar, 10 μm. The box-and-whisker plot depicts the median, quartiles, and range (Mann–Whitney *U* test). *n* = 6 mice/group. **(K)** Representative Weigert's resorcin-fuchsin-stained images of common carotid arteries (CCAs) (left) and quantification of internal elastic lamina (IEL) thickness (right) from PBS control and *B. pseudocatenulatum* mice at 24 wpfg. Scale bar, 10 μm. The box-and-whisker plot depicts the median, quartiles, and range (Mann–Whitney *U* test). *n* = 6 mice/group. **(L)** Representative immunofluorescence images of CD31 staining (left) and quantification of % area occupied with CD31 (right) in cortex, striatum, hippocampus, and substantia nigra from PBS-treated and *B. pseudocatenulatum*-treated mice at 24 wpfg. Scale bar, 50 μm. All box-and-whisker plots depict the median, quartiles, and range (Mann–Whitney *U* test). *n* = 6 mice/group. *B. pseudocatenulatum*, *Bifidobacterium pseudocatenulatum*; HE, hematoxylin and eosin; CALP, calprotectin; LF, lactoferrin; wpfg, weeks post-first gavage; VEGF, vascular endothelial growth factor; eNOS, endothelial nitric oxide synthase.

Similarly, both fecal and serum concentrations of histamine and serotonin were significantly increased in *B. pseudocatenulatum*-treated mice at 12 and 24 wpfg (Fig. 3E and F), aligning with the human metabolomics findings. Elevated serum TNF-α and IL-1β confirmed systemic inflammation in *B. pseudocatenulatum*-treated mice at 12 and 24 wpfg (Fig. 3G). Moreover, VEGF and eNOS levels were significantly increased in both serum and brain homogenates in *B. pseudocatenulatum*-treated mice compared with PBS-treated mice at 24 wpfg (Fig. 3H and I). Immunofluorescence analysis also confirmed enhanced VEGF expression in the cerebral vasculature in the *B. pseudocatenulatum*-treated mice at 24 wpfg (Fig. 3J). These results indicate that oral administration of *B. pseudocatenulatum* increases histamine and serotonin levels. Following release from the gut lumen, these microbial metabolites enter the circulation, where they act as effector molecules to drive systemic inflammation and cerebrovascular angiogenesis.

To assess vascular structural remodeling, we first evaluated the anatomy of the circle of Willis by a transcardiac injection of carbon black dye. No significant difference was observed in the structure of the major arteries at the base of the brain between *B. pseudocatenulatum*-treated mice and PBS-treated mice at 24 wpfg (Fig. S1A). Weigert's resorcin-fuchsin staining revealed thickening of the internal elastic lamina in the common carotid arteries, internal carotid arteries, and the aorta of *B. pseudocatenulatum-*treated mice at 24 wpfg compared with PBS mice (Fig. 3K; Fig. S1E and S1G). In addition, immunofluorescence staining for CD31 demonstrated increased cerebral microvessel density in the cortex, striatum, hippocampus, and substantia nigra in *B. pseudocatenulatum*-treated mice compared with PBS-treated mice at 24 wpfg (Fig. 3L). Furthermore, we evaluated vascular smooth muscle cells of the common carotid arteries with an antibody against α-smooth muscle actin (α-SMA) using immunofluorescence staining. The result showed that there was a decreasing tendency in α-SMA levels in the tunica media in *B. pseudocatenulatum*-treated mice compared with the PBS-treated mice at 24 wpfg, although the difference did not reach statistical significance (Fig. S1C). Thus, chronic administration of *B. pseudocatenulatum*—despite its conventional classification as a probiotic—elicited intestinal and systemic inflammation accompanied by pathological cerebrovascular angiogenesis, effectively recapitulating characteristic MMD-relevant vascular signatures.

### Histamine and serotonin independently reproduce MMD-relevant angiogenesis *in vivo*

To determine whether the microbial metabolites histamine and serotonin directly mediate vasculopathy, mice were intraperitoneally injected with histamine, serotonin, or PBS every other day for 12 weeks (Fig. 4A). Both histamine- and serotonin-injected groups exhibited significantly elevated levels of VEGF and eNOS in serum and brain tissue compared with PBS-injected controls (Fig. 4B and C). Immunofluorescence staining further confirmed increased VEGF expression in cerebrovascular endothelial cells of serotonin-injected and histamine-injected mice (Fig. 4D).

**Figure 4:**
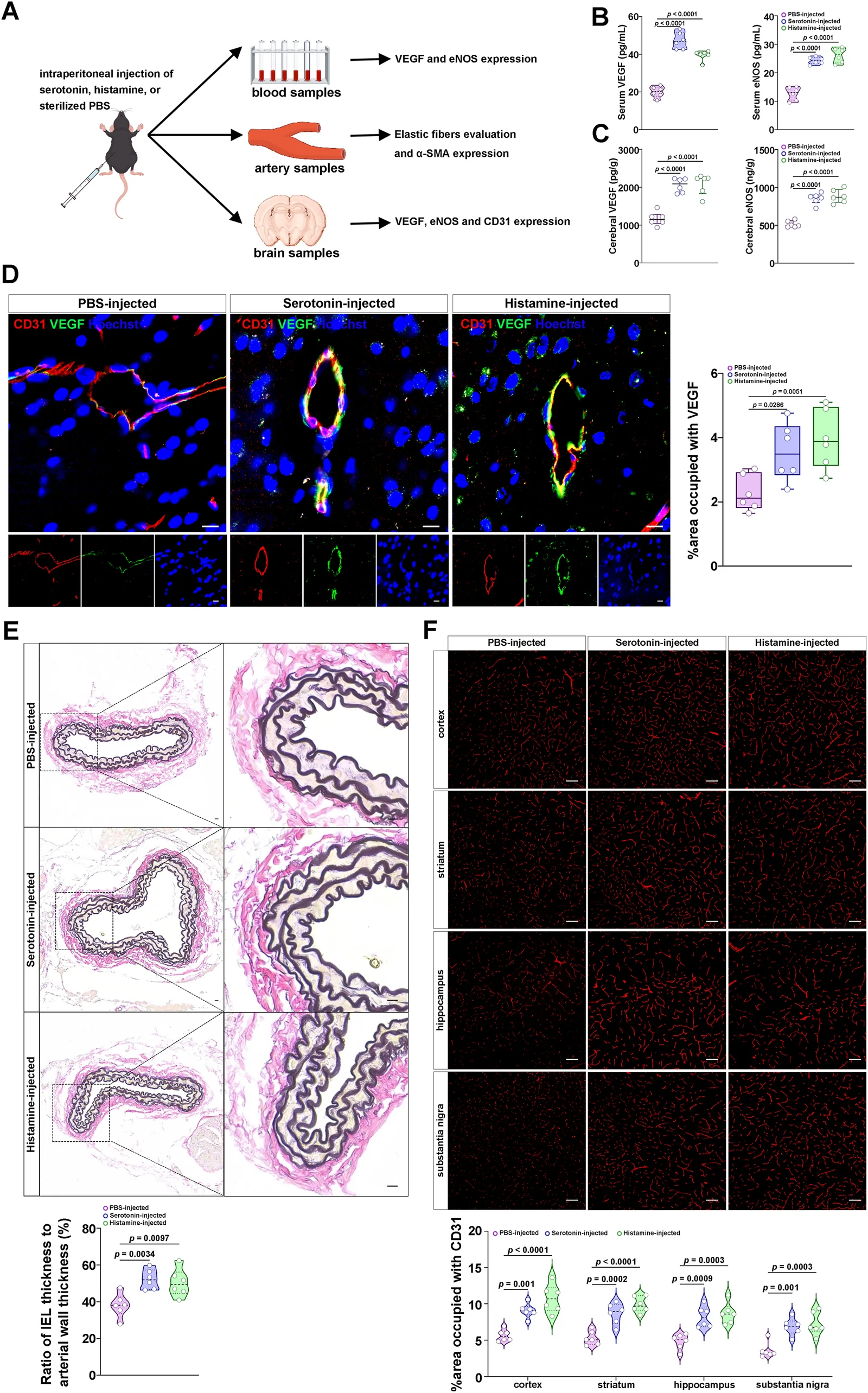
Serotonin and histamine trigger vascular remodeling. **(A)** Schematic of the experimental design for intraperitoneal serotonin or histamine injections. **(B)** ELISA analysis of serum VEGF and eNOS levels in PBS-injected, serotonin-injected, and histamine-injected mice at 12 weeks post-injection. All box-and-whisker plots depict the median, quartiles, and range (one-way ANOVA with Tukey's multiple comparisons test). *n* = 6 mice/group. **(C)** ELISA analysis of cerebral VEGF and eNOS levels in PBS-injected, serotonin-injected, and histamine-injected mice. All box-and-whisker plots depict the median, quartiles, and range (one-way ANOVA with Tukey's multiple comparisons test). *n* = 6 mice/group. **(D)** Representative immunofluorescence images (left) of VEGF (green) and CD31 (red) in brain sections, and quantification of VEGF fluorescence area % (right), among PBS-injected, serotonin-injected, and histamine-injected mice. Scale bar, 10 μm. The box-and-whisker plot depicts the median, quartiles, and range (one-way ANOVA with Tukey's multiple comparisons test). *n* = 6 mice/group. **(E)** Representative Weigert's resorcin-fuchsin-stained images of common carotid arteries (CCAs) (up) and quantification of internal elastic lamina (IEL) thickness (down) from PBS-injected, serotonin-injected, and histamine-injected mice. Scale bar, 20 μm. The box-and-whisker plot depicts the median, quartiles, and range (one-way ANOVA with Tukey's multiple comparisons test). *n* = 6 mice/group. **(F)** Representative immunofluorescence images of CD31 staining in cortex, striatum, hippocampus, and substantia nigra (up) and quantification of % area occupied with CD31 (down) in brain sections from PBS-injected, serotonin-injected, and histamine-injected mice. Scale bar, 50 μm. All box-and-whisker plots depict the median, quartiles, and range (one-way ANOVA with Tukey's multiple comparisons test). *n* = 6 mice/group. VEGF, vascular endothelial growth factor; eNOS, endothelial nitric oxide synthase.

No significant difference in the anatomy of the circle of Willis was found among PBS-injected, serotonin-injected, and histamine-injected mice, evaluated by a transcardiac injection of carbon black dye (Fig. S1B). Morphometric analysis of the common carotid arteries, internal carotid arteries, and the aorta revealed a thickened internal elastic lamina in both histamine- and serotonin-injected mice (Fig. 4E; Fig. S1F and S1H). CD31 immunostaining also demonstrated increased cerebral microvessel density in the cortex, striatum, hippocampus, and substantia nigra in both serotonin-injected and histamine-injected mice, indicating enhanced angiogenesis (Fig. 4F). Consistent with MMD-relevant medial thinning, α-SMA immunoreactivity was markedly attenuated in the tunica media of common carotid arteries following histamine or serotonin administration, suggesting a loss and/or phenotypic modulation of contractile smooth muscle cells (Fig. S1D).

Collectively, these results indicate that histamine and serotonin are sufficient to recapitulate key MMD-relevant vascular features *in vivo*. These findings provide functional evidence that *B. pseudocatenulatum*-associated metabolites are not only correlated with but functionally contribute to cerebrovascular remodeling, supporting a direct gut–brain vascular axis in MMD.

## Discussion

The gut microbiota is increasingly recognized as a key regulator of host metabolism, immune signaling, and vascular function.^27^ Dysbiosis has been implicated in cerebrovascular disorders, including intracranial aneurysms,^28^ ischemic stroke,^29^ and intracerebral hemorrhage.^30^ MMD, a rare and progressive cerebrovascular condition characterized by arterial stenosis and abnormal neovascularization, has likewise been associated with inflammation and immune dysregulation.^31^, ^32^, ^33^ Previous microbiome studies in MMD described compositional shifts,^19^^,^^20^ but lacked mechanistic validation. Thus, a central unresolved issue has been whether gut microbial alterations merely accompany MMD or actively contribute to its pathogenesis.

In this study, we address this question by integrating multi-omics profiling with *in vivo* functional validation to define a gut–brain vascular axis in MMD. We identified enrichment of *B. pseudocatenulatum*, a species usually regarded as probiotic, accompanied by elevated histamine and serotonin. Chronic oral administration of *B. pseudocatenulatum* in mice disrupted intestinal barrier integrity, increased circulating histamine/serotonin, induced systemic inflammation, and activated angiogenic pathways, facilitating cerebrovascular remodeling. Notably, direct administration of histamine or serotonin reproduced an MMD-relevant angiogenic phenotype, confirming these metabolites as sufficient effectors. Together, these findings establish a microbial–metabolite–vascular signaling axis, in which *B. pseudocatenulatum*-associated histamine and serotonin are likely to function as active mediators rather than passive byproducts. This causal chain—microbial dysbiosis leading to metabolite overproduction, systemic inflammation, and vascular remodeling—clarifies how gut bacteria can directly drive cerebrovascular pathology in MMD.

However, it remains unclear whether the elevated concentrations of histamine and serotonin directly result from the enrichment of *B. pseudocatenulatum*. The gut microbiota is an important source of gut histamine, which is derived from histidine decarboxylation under the action of histidine decarboxylase.^34^ Research has shown that *B. animalis BD400* can alleviate collagen-induced arthritis by regulating histidine metabolism. Human 5-HT is primarily produced in the intestine, with over 90% originating there, and gut bacteria influence its production by expressing tryptophan synthase.^35^ A study confirmed the promotion of serotonin secretion in enterochromaffin cells by *Bifidobacterium*.^36^ Although no studies to date have directly examined the relationship between *B. pseudocatenulatum* and histamine or serotonin production, future experiments should determine whether the elevated concentrations of these metabolites directly result from enrichment of this species.

Our study presents a comprehensive characterization of the gut microbiota in MMD patients, revealing a striking and unexpected enrichment of the genus *Bifidobacterium*, particularly *B. pseudocatenulatum*. This finding is particularly notable given that *Bifidobacterium* species are generally regarded as beneficial probiotics.^37^ While probiotics have demonstrated positive effects on gut health, accumulating evidence, including a clinical trial showing increased mortality with probiotic treatment in acute pancreatitis,^38^ highlights potential risks of their indiscriminate use.^39^^,^^40^ Previous reports suggest that *B. pseudocatenulatum* can enhance gut integrity and reduce inflammation, which appears contradictory to our findings.^41^, ^42^, ^43^ However, those studies were conducted under different disease contexts, whereas our study examined effects in an otherwise healthy setting. Our findings demonstrate that, under specific pathological conditions, even traditionally beneficial microbes can become disease-promoting pathobionts. This highlights the need for careful evaluation of probiotic interventions, especially in neurologically vulnerable populations. In addition to *B. pseudocatenulatum*, other enriched taxa, such as *Firmicutes bacterium CAG:227*, were observed in MMD. We initially detected an increase in *Bifidobacterium* abundance using 16S rDNA sequencing and subsequently refined this signal through shotgun metagenomic analysis, ultimately identifying *B. pseudocatenulatum* as the dominant species. This analytical progression explains why we focused primarily on *B. pseudocatenulatum* rather than other enriched taxa, including *Firmicutes bacterium CAG:227*.

Mechanistically, our metabolomics data revealed activation of the tryptophan pathway in MMD patients, marked by elevated serotonin. This is consistent with prior studies showing that certain *Bifidobacterium* strains stimulate gut-derived serotonin production.^44^^,^^45^ Although serotonin is essential for gut homeostasis, excessive levels have been implicated in inflammation and pathological angiogenesis.^46^ Serotonin promotes endothelial cell proliferation^47^^,^^48^ and migration via specific receptors such as 5-HT_2_B, activating downstream pro-angiogenic pathways including VEGF signaling.^49^^,^^50^ In our study, serotonin injection alone replicated key features of MMD vasculopathy, confirming its role as an effector metabolite. Similarly, histamine—while essential for immunomodulation and neurotransmission^51^, ^52^, ^53^—has been implicated in aberrant angiogenesis in inflammatory and tumor settings.^54^^,^^55^ Histamine promotes endothelial proliferation and VEGF production, particularly when synergizing with growth factors such as bFGF.^56^^,^^57^ Our *in vivo* findings showed that histamine injection induced elevated VEGF levels and cerebrovascular remodeling, supporting its contribution to MMD pathogenesis.

We observed elevated fecal and circulating levels of histamine and serotonin, metabolites that are closely associated with allergic responses. Previous studies have shown that *Ruminococcus gnavus* is increased in patients with MMD,^19^ an increase that was strongly associated with food and respiratory allergy, through the promotion of IL-5 and IL-13 secretion from lymphocytes.^58^ In addition, Mineharu et al reported that increased lipid-leukocyte modules in the peripheral blood enhanced the penetrance of the *RNF213 R4810K* mutation and further impacted clinical severity in MMD.^59^ These lipid-leukocyte modules include GATA2, a regulator of eosinophil or mast cell differentiation;^60^ together, the above studies also imply an association between MMD and allergy. Consistent with these findings, comprehensive molecular analyses using an autoimmune-related gene predictive model and immune infiltrations based on machine learning methods in MMD, as well as vascular transcriptome data from MMD patients, support a model of eosinophilic and type-2 inflammation.^61^ All the above evidence indicates that MMD is closely associated with allergy, which also indirectly supports our conclusions that histamine and serotonin are involved in MMD-relevant vascular phenotypes.

A notable divergence in findings emerged when compared with a previous report by Yu et al, which described reduced levels of *Bifidobacterium* in MMD patients.^19^^,^^20^ Several factors may account for this discrepancy. First, our cohort was restricted to patients with angiographic stage 2–3 disease, whereas the study by Yu et al did not stratify by disease severity—an important determinant of microbiome composition.^62^ Second, our controls were cohabiting individuals, minimizing environmental and dietary variability; by contrast, Yu et al used general population controls. Third, our participants were recruited from a geographically consistent region (South and Central China), whereas prior studies did not account for regional microbiota variation. These differences suggest that regional and disease-stage-specific microbiome patterns may underlie the contrasting results.

Furthermore, there are reports of a higher abundance of *Campylobacter* in the oral microbiome of Japanese patients with MMD.^63^ *Campylobacter* can drive intestinal inflammation, impair the intestinal barrier, and prime the intestine for chronic inflammatory responses, in part by altering the composition of the intestinal microbiota. This is consistent with our findings that the differential bacterial species can result in intestinal inflammation. Together, these observations highlight the importance of rigorous cohort design and geographic consideration in microbiome studies of rare diseases such as MMD.

The innovation and significance of this work are threefold. First, we demonstrate that a conventionally probiotic species can act as a context-dependent pathobiont, overturning the assumption that all bifidobacteria are beneficial.^37^ Second, we identify histamine and serotonin as causal intermediates that link microbial activity to cerebrovascular remodeling, providing a mechanistic framework for understanding angiogenesis in neurovascular disease. Third, by completing the loop from human cohort data to oral bacterial administration and metabolite supplementation, our study offers a conceptual blueprint for dissecting microbiota–metabolite–host axes in other conditions.

From a clinical perspective, our findings open several translational avenues. First, measurement of fecal or circulating histamine and serotonin could serve as noninvasive biomarkers to identify patients at risk of MMD progression or poor surgical outcomes. Second, microbiome-targeted therapies—including selective probiotic restriction, bacteriophage-based depletion, or inhibition of microbial histidine/tryptophan decarboxylases—may represent novel preventive or adjunctive strategies. Third, existing histamine and serotonin receptor antagonists, already widely used in allergy and psychiatric practice, may warrant repurposing trials to attenuate pathological angiogenesis in MMD. Given that MMD often manifests in children and young adults, early-life microbiome monitoring and metabolite profiling may enable preventive strategies in high-risk families. Notably, histamine (H1/H2) and serotonin (5-HT_2_B) receptor antagonists are already in clinical use for allergy, gastroesophageal reflux, and psychiatric disorders. Repurposing trials could accelerate therapeutic translation without requiring new drug development. Collectively, these strategies underscore the potential for microbiome-informed precision medicine in MMD.

Notably, we observed that α-SMA immunofluorescence appeared weaker in the *B. pseudocatenulatum*-treated group, whereas Weigert's resorcin-fuchsin staining indicated increased thickness of the elastic lamina/arterial wall in some internal carotid artery segments. These findings are not necessarily inconsistent, because the two staining methods reflect different components of the vascular wall. α-SMA primarily labels vascular smooth muscle cells and may indicate changes in their abundance or contractile phenotype, whereas Weigert's staining reflects remodeling of elastic fibers and arterial wall structure. Thus, decreased α-SMA immunoreactivity may coexist with thickening of the elastic lamina or vessel wall during vascular remodeling. The non-uniform thickening observed in only some arterial segments further suggests spatial heterogeneity in vascular injury, which may be influenced by local hemodynamic stress, inflammatory responses, disease stage, or sampling orientation. As this study was not designed to assess segment-specific arterial heterogeneity, this issue should be interpreted cautiously and warrants further investigation in future studies.

This study has several limitations. First, we did not provide direct evidence that *B. pseudocatenulatum* abundance relates to fecal histamine or serotonin in the human cohort, and did not directly identify the microbial enzymes—such as histidine or tryptophan decarboxylase—responsible for histamine and serotonin production. Increased circulating histamine and serotonin levels were found in the MMD group, which provides indirect evidence supporting our selection of histamine and serotonin as the primary metabolites of interest. Second, our clinical cohort was restricted to South and Central China, and potential confounding factors such as dietary habits, medication use, and allergy-related status, which may affect gut microbial composition and histamine- and serotonin-related metabolism, were not systematically collected or controlled in the present study, underscoring the need for replication in larger, multi-ethnic populations. Third, we did not perform longitudinal stool microbiota profiling in the mouse experiments and therefore cannot formally demonstrate stable engraftment over time. In addition, the current study does not define the precise molecular signaling mechanisms by which these microbial metabolites induce intimal hyperplasia and smooth muscle cell loss (*e.g.*, endothelial activation, inflammatory signaling cascades, or smooth muscle phenotypic switching). Fourth, current data support that histamine/serotonin are sufficient to promote angiogenic/vascular changes, but do not show they are necessary for the phenotype induced by *B. pseudocatenulatum*. Moreover, the animal model did not fully reproduce the hallmark pathological features of MMD, particularly stenosis of the terminal internal carotid arteries and the development of typical moyamoya vessels, and therefore is not synonymous with the full human disease phenotype. Thus, caution is warranted when extrapolating these findings to the human disease. Despite these limitations, our findings provide compelling evidence for a functional gut–brain axis in MMD. We demonstrate that expansion of *B. pseudocatenulatum* promotes systemic elevation of histamine and serotonin, which is sufficient to induce pathological angiogenesis characterized by intimal hyperplasia and smooth muscle cell loss. This multi-level validation—from microbial dysbiosis to metabolite-driven vascular remodeling—establishes a causal framework that positions these metabolites as active pathogenic effectors rather than passive correlates. Future studies should prioritize defining the specific metabolite–receptor interactions and downstream signaling cascades that orchestrate this neurovascular transformation. Elucidation of these molecular nodes will be essential for translating our findings into clinical interventions, including targeted pharmacological blockade and precision microbiome-based therapies. Collectively, these efforts will be critical for consolidating and generalizing the proposed mechanistic framework and for accelerating its translation into clinical practice.

In summary, our findings define a gut–brain vascular axis in MMD, wherein *B. pseudocatenulatum*-driven dysbiosis promotes histamine- and serotonin-mediated inflammation and angiogenesis. This work provides a mechanistic foundation for understanding MMD pathogenesis, highlights the potential risks associated with unsupervised probiotic use, and identifies microbial metabolites as promising diagnostic and therapeutic targets for precision medicine approaches.

## CRediT authorship contribution statement

**Haiyan Tian:** Writing – original draft, Project administration, Methodology, Investigation, Formal analysis. **Lilan Zhu:** Software, Project administration, Methodology. **Yuheng Zhang:** Visualization, Project administration, Methodology. **Mengwei Chen:** Visualization, Project administration, Formal analysis. **Xueying Zuo:** Visualization, Software, Project administration. **Yi Wu:** Project administration, Methodology, Data curation. **Hanzhen Wang:** Software, Methodology. **Zheng Lv:** Validation, Project administration, Methodology. **Qing Huang:** Validation, Supervision, Funding acquisition. **Min Wu:** Writing – review & editing. **Erxi Wu:** Writing – review & editing, Supervision, Resources, Formal analysis. **Jinsheng Zeng:** Visualization, Validation, Supervision, Funding acquisition, Data curation. **Xuejing Wang:** Writing – original draft, Supervision, Investigation, Funding acquisition, Formal analysis, Conceptualization.

## Ethics declaration

This study was approved by the Institutional Ethics Committees of the First Affiliated Hospital of Zhengzhou University (No. 2021-KY-0848-002). All participants provided informed consent.

## Data availability

The data collected in this study, including de-identified participant data and the data dictionary, are available to researchers through the corresponding author, Prof. Xuejing Wang, upon reasonable request. These data will be available for three years after publication. Data requests require a methodologically sound proposal as well as a data access agreement and approval by the local ethics committee.

## Funding

This work was supported by the following funding agencies: the 10.13039/501100001809National Natural Science Foundation of China (No. 82122022, 82471272, 82171248, 82271365), Talent Program of the First Affiliated Hospital of 10.13039/501100002402Sun Yat-sen University (China) (No. R04017), Application Form for Hundred Talents Program of 10.13039/501100002402Sun Yat-sen University (China) (No. R03027), Henan Scientific and Technological Development Program (China) (No. 232301420015), the 10.13039/501100001809Natural Science Foundation of China (key program: No. 82130035; general program: No. 82371308), and Guangdong Provincial Clinical Research Center for Neurological Diseases (China) (No. 2020B1111170002).

## Conflict of interests

Erxi Wu is a member of *Genes & Diseases* Editorial Board. To minimize bias, he was excluded from all editorial decision-making related to the acceptance of this article for publication. The remaining authors declare no competing interests.
