# Supplementary material for: Gut microbial histamine and serotonin promote cerebrovascular angiogenesis: Evidence from moyamoya disease
Source: Genes Dis. 2026 Jun 13;14(2):102306. doi: 10.1016/j.gendis.2026.102306 (PMC13684997; doi:10.1016/j.gendis.2026.102306)
Supplement: Supplementary file 1 [file mmc1.docx]

**SUPPLEMENTARY MATERIALS**

This file provides supporting data referenced in the manuscript, “Gut microbial histamine and serotonin promote cerebrovascular angiogenesis: evidence from moyamoya disease.” It includes:

1. **Alpha diversity metrics derived from 16S rDNA and shotgun metagenomic sequencing**
2. **Genus- and species-level differential abundance analyses**
3. **A comprehensive assessment of the intracranial and systemic vascular remodeling in murine models**

These results reinforce the gut microbiota dysbiosis observed in Moyamoya disease (MMD) and highlight the enrichment of *B. pseudocatenulatum* as a key taxonomic signature. In addition, the murine data demonstrate MMD-relevant cerebrovascular remodeling following chronic microbial colonization or metabolite administration.

**Supplementary Table S1. Alpha diversity metrics comparing normal controls (NC) and MMD groups based on 16S rDNA sequencing.**

| **Alpha diversity** | **NC (n = 49)** | **MMD (n = 79)** | ***p* value** |
| --- | --- | --- | --- |
| **Sob** | 248.0 (181.5 – 297.5) | 241.0 (181.0 – 308.0) | 0.6595 |
| **Chao1** | 294.0 (212.4 – 357.7) | 286.7 (210.5 – 355.0) | 0.6426 |
| **Shannon** | 4.602 (3.841 – 5.106) | 4.704 (4.182 – 5.012) | 0.5221 |
| **Simpson** | 0.9096 (0.8390 – 0.9407) | 0.9155 (0.8711 – 0.9355) | 0.4546 |
| **ACE** | 296.7 (213.6 – 362.7) | 285.1 (217.4 – 365.1) | 0.7105 |
| **Goods’ coverage** | 0.9992 (0.9990 – 0.9994) | 0.9992 (0.9990 – 0.9994) | 0.3429 |
| **Pielou** | 0.5867 (0.5058 – 0.6343) | 0.5865 (0.5561 – 0.6372) | 0.2033 |
| **PD** | 28.07 (22.26 – 35.07) | 27.49 (21.51 – 32.66) | 0.6356 |

Note. Data are presented as median (interquartile range). *P* values were calculated using the Mann–Whitney U test. NC, normal controls; MMD, moyamoya disease.

**Supplementary Table S2. Relative abundance of genera between NC and MMD groups based on the 16S rDNA sequencing data**.

| **Genus** | **NC (n = 49)** | **MMD (n = 79)** | ***p* value** |
| --- | --- | --- | --- |
| ***Bacteroides*** | 4.951 (1.135 – 14.66) | 6.873 (1.834 – 16.94) | 0.4429 |
| ***Escherichia-Shigella*** | 0.4099 (0.0807 – 2.050) | 0.9359 (0.1659 – 3.731) | 0.1402 |
| ***Prevotella_9*** | 0.3149 (0.0000 – 9.8740) | 0.0148 (0.000 – 8.370) | 0.5072 |
| ***Faecalibacterium*** | 4.266 (2.558 – 8.205) | 5.371 (2.147 – 11.97) | 0.4321 |
| ***Megamonas*** | 0.0036 (0.000 – 19.92) | 0.0045 (0.000 – 1.722) | 0.6220 |
| ***Bifidobacterium*** | 0.7614 (0.2082 – 2.406) | 2.233 (0.5007 – 8.299) | **0.0046** |
| ***Agathobacter*** | 3.555 (0.9968 – 7.441) | 2.729 (0.8729 – 6.226) | 0.4480 |
| ***Subdoligranulum*** | 1.806 (0.8062 – 5.107) | 2.074 (0.4501 – 5.035) | 0.5617 |
| ***Blautia*** | 2.086 (0.9579 – 5.449) | 2.416 (1.339 – 4.903) | 0.5158 |
| ***Dialister*** | 0.5559 (0.0033 – 3.118) | 0.0110 (0.0017 – 2.777) | 0.2422 |
| ***Phascolarctobacterium*** | 0.5786 (0.0036 – 4.575) | 0.4141 (0.0199 – 2.875) | 0.9834 |
| ***Megasphaera*** | 0.000 (0.000 – 0.6397) | 0.0016 (0.000 – 1.159) | 0.2768 |
| ***Streptococcus*** | 0.2593 (0.0806 – 0.9785) | 0.1587 (0.0573 – 0.7516) | 0.1892 |
| ***Collinsella*** | 0.6188 (0.2165 – 1.499) | 1.112 (0.3351 – 2.712) | 0.1157 |
| ***Eubacterium_coprostanoligenes_group*** | 0.6055 (0.0384 – 2.209) | 0.6712 (0.0642 – 1.826) | 0.7609 |
| ***Ruminococcus_2*** | 0.4050 (0.0016 – 1.736) | 0.7021 (0.0017 – 2.742) | 0.5390 |
| ***Lachnoclostridium*** | 0.7914 (0.4890 – 2.312) | 0.8904 (0.3893 – 1.815) | 0.7509 |
| ***Lactobacillus*** | 0.0255 (0.0088 – 0.7657) | 0.0554 (0.0035 – 0.3266) | 0.7204 |
| ***Klebsiella*** | 0.0569 (0.0050 – 0.7401) | 0.0698 (0.0051 – 0.7868) | 0.9347 |
| ***Ruminococcaceae_UCG-002*** | 0.3882 (0.0090 – 1.548) | 0.2910 (0.0092 – 1.389) | 0.7475 |

Note. Data are presented as median (interquartile range). *P* values were calculated using the Mann–Whitney U test. Bold values indicate statistical significance (*p* < 0.05). NC, normal controls; MMD, moyamoya disease.

**Supplementary Table S3. The α-diversity index between NC and MMD groups based on the metagenome sequencing data.**

| **Alpha diversity** | **NC (n = 30)** | **MMD (n = 30)** | ***p* value** |
| --- | --- | --- | --- |
| **Observed species** | 2833 (2317 – 3622) | 2699 (2297 – 3393) | 0.4969 |
| **Shannon** | 6.319 (6.005 – 6.830) | 6.367 (5.915 – 6.600) | 0.7524 |
| **Simpson** | 0.9600 (0.9303 – 0.9698) | 0.9611 (0.9368 – 0.9675) | 0.9357 |
| **Chao1** | 2936 (2379 – 3710) | 2806 (2400 – 3509) | 0.5422 |
| **ACE** | 2916 (2374 – 3701) | 2793 (2366 – 3492) | 0.4853 |

Note. Data presented as median (interquartile range). *P* values were calculated using the Mann–Whitney U test. NC, normal controls; MMD, moyamoya disease.

**Supplementary Table S4. Relative abundance of species between NC and MMD groups based on the metagenome sequencing data**.

| **Species** | **NC (n = 30)** | **MMD (n = 30)** | ***p* value** |
| --- | --- | --- | --- |
| ***Bifidobacterium longum*** | 0.0034 (0.0003 – 0.0092) | 0.0065 (0.0031 – 0.0105) | 0.0772 |
| ***Faecalibacterium prausnitzii*** | 0.01485 (0.0039 – 0.0310) | 0.0250 (0.0085 – 0.0546) | 0.1462 |
| ***Clostridium sp. CAG:452*** | 4.98 × 10^-6^ (4.86 × 10^-7^ – 3.848 × 10^-5^) | 4.42 × 10^-6^ (4.68 × 10^-7^ – 6.19 × 10^-5^) | 0.8399 |
| ***Escherichia coli*** | 0.0009 (0.0003 – 0.0046) | 0.0016 (0.0001 – 0.0034) | 0.8315 |
| ***Lactobacillus salivarius*** | 3.355 × 10^-5^ (5.618 × 10^-6^ – 0.0002) | 1.825 × 10^-5^ (9.363 × 10^-6^ – 8.134 × 10^-5^) | 0.9678 |
| ***Firmicutes bacterium CAG:227*** | 0.0002 (7.265 × 10^-5^ – 0.0010) | 0.0006 (0.0002 – 0.0030) | **0.0225** |
| ***Eubacterium sp. CAG:180*** | 7.145 × 10^-5^ (1.48 × 10^-5^ – 0.0041) | 3.15 × 10^-5^ (4.725 × 10^-6^ – 0.0026) | 0.3335 |
| ***[Eubacterium] rectale*** | 0.0034 (0.0017 – 0.0191) | 0.0047 (0.0020 – 0.0141) | 0.6973 |
| ***Romboutsia timonensis*** | 0.0016 (0.0001 – 0.0040) | 0.0021 (0.0009 – 0.0067) | 0.2665 |
| ***Bifidobacterium pseudocatenulatum*** | 0.0005 (9.953 × 10^-5^ – 0.0022) | 0.0107 (0.0055 – 0.0233) | **< 0.0001** |
| ***Prevotella copri*** | 0.0007 (1.67 × 10^-5^ – 0.0083) | 1.39 × 10^-5^ (3.468 × 10^-6^ – 0.0006) | **0.0227** |
| ***Dialister succinatiphilus*** | 6.795 × 10^-6^ (1.175 × 10^-6^ – 4.843 × 10^-5^) | 1.135 × 10^-5^ (3.718 × 10^-6^ – 0.0002) | 0.5520 |
| ***[Clostridium] hiranonis*** | 4.08 × 10^-6^ (1.021 × 10^-6^ – 1.158 × 10^-5^) | 7.245 × 10^-6^ (3.873 × 10^-6^ – 2.008 × 10^-5^) | 0.0532 |
| ***Bifidobacterium adolescentis*** | 0.0025 (0.0002 – 0.01416) | 0.0048 (0.0025 – 0.0121) | 0.2794 |
| ***Eubacterium sp. CAG:202*** | 6.760 × 10^-5^ (1.888 × 10^-5^ – 0.0006) | 0.0001 (3.288 × 10^-5^ – 0.0004) | 0.4232 |
| ***Roseburia hominis*** | 0.0005 (0.0003 – 0.0010) | 0.0004 (0.0003 – 0.0013) | 0.5039 |
| ***Oscillibacter sp. CAG:241*** | 2.995 × 10^-5^ (1.175 × 10^-5^ – 0.0003) | 6.72 × 10^-5^ (1.1 × 10^-5^ – 0.0002) | 0.6892 |
| ***Holdemanella biformis*** | 0.0002 (5.228 × 10^-5^ – 0.0023) | 0.0002 (0.0001 – 0.0019) | 0.5325 |
| ***Bacteroides coprocola*** | 6.885 × 10^-5^ (3.475 × 10^-6^ – 0.0007) | 2.545 × 10^-5^ (4.42 × 10^-6^ – 9.335 × 10^-5^) | 0.2301 |
| ***Clostridium sp. CAG:302*** | 7.185 × 10^-7^ (0.000 – 4.283 × 10^-5^) | 2.77 × 10^-7^ (0.000 – 0.0002) | 0.9553 |

Note. Data presented as median (interquartile range). *P* values were calculated using the Mann–Whitney U test. Bold values indicate statistical significance (*p* < 0.05). NC, normal controls; MMD, moyamoya disease.

**Supplementary Fig. 1 Comprehensive assessment of the intracranial and systemic vasculature. A,** Gross anatomy of the major arteries at the base of the brain assessed by transcardiac carbon black dye injection in PBS-treated and *Bifidobacterium pseudocatenulatum–*treated mice (*B. pseudocatenulatum*–treated mice) at 24 weeks post–first gavage (wpfg). **B,** Gross anatomy of the major arteries at the base of the brain assessed by transcardiac carbon black dye injection in PBS-injected, serotonin-injected, and histamine-injected mice. **C,** Representative immunofluorescence images of α-smooth muscle actin (α-SMA) staining and quantification of mean fluorescence intensity in common carotid arteries (CCAs) from PBS-treated and *B. pseudocatenulatum*–treated mice at 24 wpfg. Scale bar, 50 μm. The box-and-whisker plot depicts the median, quartile, and range (Mann–Whitney U test). n = 6 mice/group. **D,** Representative immunofluorescence images of α-SMA staining and quantification of mean fluorescence intensity in CCAs from PBS-injected, serotonin-injected, and histamine-injected mice. Scale bar, 50 μm. The box-and-whisker plot depicts the median, quartiles and range (one-way ANOVA with Tukey’s multiple-comparisons test). n = 6 mice/group. **E,** Representative Weigert’s resorcin–fuchsin–stained images of internal carotid arteries (ICAs) (left) and quantification of internal elastic lamina (IEL) thickness (right) in PBS-treated and *B. pseudocatenulatum*–treated mice at 24 wpfg. Scale bar, 50 μm. The box-and-whisker plot depicts the median, quartiles and range (Mann–Whitney U test). n = 6 mice/group. **F,** Representative Weigert’s resorcin–fuchsin–stained images of ICAs (left) and quantification of IEL thickness (right) in PBS-injected, serotonin-injected, and histamine-injected mice. Scale bar, 50 μm. The box-and-whisker plot depicts the median, quartiles and range (one-way ANOVA with Tukey’s multiple-comparisons test). n = 6 mice/group. **G,** Representative Weigert’s resorcin–fuchsin–stained images of the aorta (left) and quantification of IEL thickness (right) in PBS-treated and *B. pseudocatenulatum*–treated mice at 24 wpfg. Scale bar, 100 μm. The box-and-whisker plot depicts the median, quartiles and range (Mann–Whitney U test). n = 6 mice/group. **H,** Representative Weigert’s resorcin–fuchsin–stained images of the aorta (left) and quantification of IEL thickness (right) in PBS-injected, serotonin-injected, and histamine-injected mice. Scale bar, 100 μm. The box-and-whisker plot depicts the median, quartiles and range (one-way ANOVA with Tukey’s multiple-comparisons test). n = 6 mice/group.

**Supplementary Summary:**

These data collectively confirm:

1. No significant difference in alpha diversity between groups
2. Significant enrichment of *Bifidobacterium* at the genus level by 16S rDNA sequencing and of *B. pseudocatenulatum* at the species level by metagenomic sequencing.
3. Decreased abundance of *Prevotella copri* in MMD
4. Chronic administration of *B. pseudocatenulatum,* as well as histamine or serotonin injection, reproduces MMD-relevant vascular phenotypes in vivo.

These microbial shifts correlate with the elevated histamine and serotonin levels described in the main manuscript and support the identification of *B. pseudocatenulatum* as a context-specific pathobiont.
